# Biomechanical modeling of actively controlled rectus extraocular muscle pulleys

**DOI:** 10.1038/s41598-022-09220-x

**Published:** 2022-04-06

**Authors:** Qi Wei, Bassam Mutawak, Joseph L. Demer

**Affiliations:** 1 Department of Bioengineering, George Mason University, Suite 3100, Peterson Hall, 4400 University Drive, 1J7, Fairfax, VA 22030 USA; 2 Department of Computer Science, George Mason University, Fairfax, VA USA; 3 Department of Ophthalmology, Stein Eye Institute, University of California, Los Angeles, CA USA; 4 Bioengineering Department, University of California, Los Angeles, CA USA; 5 Neuroscience Interdepartmental Program, University of California, Los Angeles, CA USA; 6 Department of Neurology, University of California, Los Angeles, CA USA

**Keywords:** Biomedical engineering, Computational science

## Abstract

The Active Pulley Hypothesis (APH) is based on modern functional anatomical descriptions of the oculomotor plant, and postulates behaviors of the orbital pulleys proposed to be positioned by the extraocular muscles (EOMs). A computational model is needed to understand this schema quantitatively. We developed and evaluated a novel biomechanical model of active horizontal rectus pulleys. The orbital (OL) and global (GL) layers of the horizontal rectus EOMs were implemented as separate musculoskeletal strands. Pulley sleeves were modeled as tube-like structures receiving the OL insertion and suspended by elastic strands. Stiffnesses and orientations of pulley suspensions were determined empirically to limit horizontal rectus EOM side-slip while allowing anteroposterior pulley travel. Independent neural drives of the OL greater than GL were assumed. The model was iteratively refined in secondary gazes to implement realistic behavior using the simplest mechanical configuration and neural control strategy. Simulated horizontal rectus EOM paths and pulley positions during secondary gazes were consistent with published MRI measurements. Estimated EOM tensions were consistent with the range of experimentally measured tensions. This model is consistent with postulated bilaminar activity of the EOMs, and the separate roles of the GL in ocular rotation, and OL in pulley positioning.

## Introduction

The oculomotor periphery was formerly regarded as a simple structure that executed complex behaviors by explicitly specified motor commands from the central nervous system. It is now recognized that three-dimensional (3-D) eye orientation is substantially constrained by ocular motor plant mechanics because of the connective tissue pulley apparatus, which is a system of connective tissues including collagenous sleeves in posterior Tenon’s fascia, stiffened by elastin and smooth muscle^1^, that constitute a nested gimbal arrangement for the globe^1^. Magnetic resonance imaging (MRI) evidence suggesting existence of pulleys^2,3^ was a major motivation for anatomical re-examination of the tissues using modern methods^4–9^ that changed understanding of the functional anatomy of the orbit. The mechanically critical pulleys are proposed to stabilize paths of the extraocular muscles (EOMs) regardless of the eye’s rotational orientation, serve as the functional origins of the EOMs, and determine ocular kinematics^5^. Orbital tissues also interconnect as a ring that mechanically intercouples pulleys of the neighboring EOMs, forming an intricate gimbal system^1^.

Microscopy demonstrates that each rectus EOM is subdivided into a global layer (GL) and an orbital layer (OL)^10–12^. The GL passes through the EOM’s pulley ring, anterior to which the GL becomes tendinous and inserts on the sclera to exert oculorotary force. The OL inserts on the connective tissues of the pulley ring itself; it is proposed that this insertion permits the OL to shift the pulley anteroposteriorly in coordination with the GL insertion. The transverse positions of the pulleys are stabilized by suspensory connective tissues and intermuscular couplings.

Supported by MRI^4,13^, neurophysiological^14^, biomechanical^15,16^, and histological evidence^10,11^, the Active Pulley Hypothesis (APH) presumes important interactions of EOMs with their pulleys. The APH proposes that Listing’s law of ocular torsion is implemented mechanically by anteroposterior positioning of the pulleys through active contraction of by the OLs of the rectus EOMs^13^. Specifically, the APH posits that active OL tension, balanced against passive elastic forces in the pulley suspensions, maintains a constant distance between each rectus pulley and its corresponding scleral insertion over the entire range of ocular rotations. Consistent with selective electromyographic observations in the OL and GL of human EOMs^17^, APH posits that contractile activity in the GL must differ from that in OL of that same EOM, and that resulting differential muscle force in the two layers produces different mechanical effects: the GL rotates the eye, while the OL translates associated pulley tissues^13^. The APH requires differential force generation in the OL and GL of the same EOM. Some have claimed based on theoretical^18^ or ultrastructural grounds that fibers in the same EOM cannot be mechanically independent^19^, although even that anatomical claim is disputed by tracing of single EOM fibers in humans^12^. Recent in vitro functional studies have directly demonstrated that both passive and active tensile forces in the GL and OL of the same bovine rectus EOM may be highly independent^15,16^. Because there is currently no way to directly measure tensions separately the two layers of physiologically functioning human EOMs in vivo, a modeling approach may shed light on the likely necessity of the differential functions of the OL and GL as postulated in the APH.

To demonstrate that it is mechanically plausible, or even necessary, for the rectus GL and OL to have different tensions when pulleys behave consistently with the APH, we implemented an anatomically realistic computational model of mechanics of the eye, associated connective tissue pulleys, and bilaminar EOMs, constrained as far as possible by known anatomy and such sparse mechanical and electrophysiological measurements as currently exist. Model simulations were then used to simulate ocular rotations, for which tensions in the GL, OL, and connective tissue suspensions were compared with existing whole EOM tension data.

Modeling of pulley biomechanics has been largely neglected heretofore. Even the few attempts made notable kinematic simplifications and did not model pulley forces at all^20–24^. Previously we developed the first neuro-biomechanical orbit model capable of simulating dynamic eye movement in 3-D^25^. Two simplifications made by our previous model limited its application for examining functions of actively controlled pulleys. First, the model did not implement the GL and OL separately, and thus neglected their putative differential functions. Second, the pulleys were modeled by 1-D prismatic joints, which unrealistically simplified the pulley travel. Both simplifications were avoided in the new model presented here. Separate GL and OL were modeled, and pulleys were realistically modeled by a pulley tube structure.

In the current paper, we present a model implementing realistic mechanics of the horizontal rectus EOM pulleys to examine behavior for secondary gazes, which constitute single axis horizontal or vertical eye rotations. Through empirical iterations, we identified the necessary modeling components to realistically simulate pulley mechanics, which consist of a pulley tube structure, and suspensions to couple the tube to GL and OL. Kinematic behaviors of the model were compared to experimental data. Horizontal rectus EOM and pulley forces were examined to assess the model’s realism.

## Results

**Figure 1:**
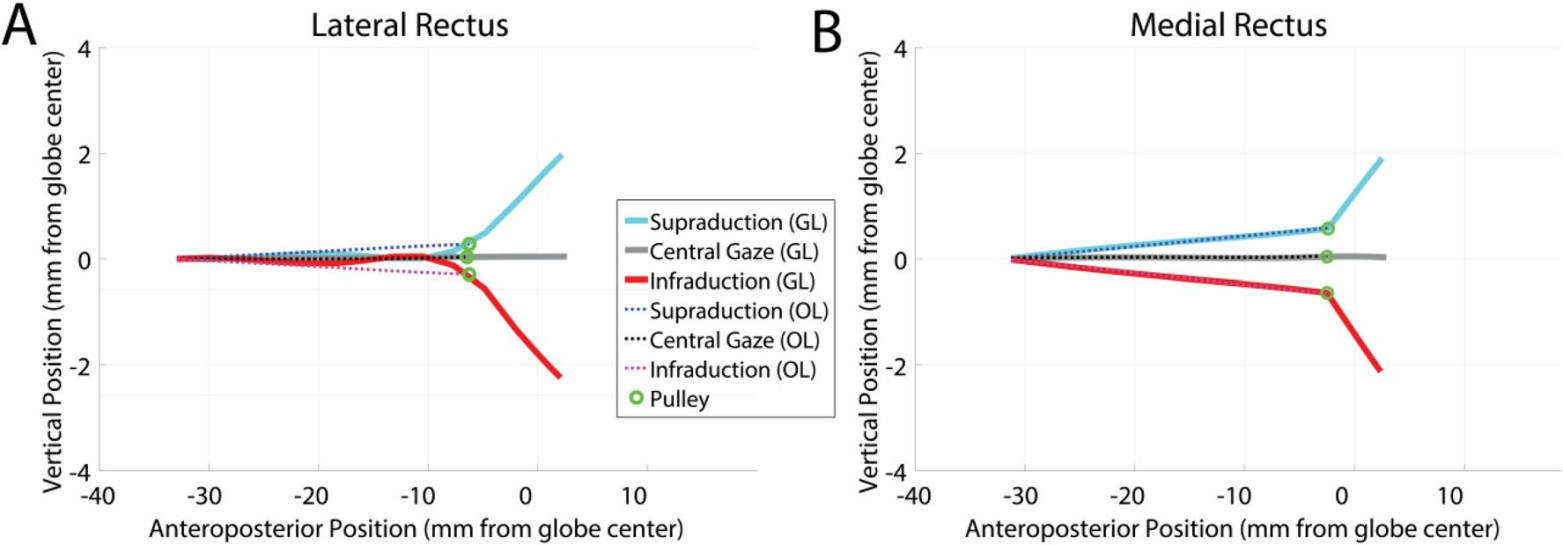
Sagittal plane visualization of simulated paths of the (**A**) lateral and (**B**) medial rectus muscle orbital and global layers in supraduction, central gaze, and infraduction. Muscle paths are inflected at the pulleys, but exhibit small transverse displacement within physiological range measured empirically by MRI.

We first examined kinematic behaviors of the new pulley model. Any rectus pulleys, whether active or not, are required to stabilize medial rectus (MR) and lateral rectus (LR) posterior paths during vertical ductions to permit what MRI demonstrates to be only small sideslip in the vertical direction^4,5^. Figure 1 shows simulated LR and MR paths in 30° supraduction, central gaze, and 30° infraduction. The model demonstrated posterior GL and OL paths to be transversely stable during vertical duction. LR pulley position, evaluated at the insertion of the OL on the pulley tube, sideslipped by less than 0.4 mm in 30° supraduction or infraduction, qualitatively consistent with empirical measurement by MRI in humans^4^.

Comparisons of simulated LR and MR sideslips with experimental data are presented in Table 1. The LR GL path showed a clear inflection near the LR pulley position, reflecting the known pulley’s role on serving as the EOM’s functional origin. The MR pulley exhibited less than 0.8 mm sideslip in the direction of vertical duction, also consistent with the empirical measurement. The MR GL path inflection occurred at the MR OL insertion, and was posterior to the globe center. Notice that there was little anteroposterior movement of the LR and MR pulleys during vertical gaze changes, so that the LR and MR anterior paths were inflected by about half the gaze angle; this is necessary and sufficient, without any contribution from oblique EOMs, to implement Listing’s law in the secondary positions of supraduction and infraduction^1^.

**Table 1:** Comparison between simulated transverse pulley shifts (mm) in secondary vertical gazes and those measured empirically from MRI data (Clark et al.^4^).

	Supraduction	Infraduction
**LR**		
Empirical	− 1.00	0.50
Simulated	0.30	− 0.26
**MR**		
Empirical	0.40	0.00
Simulated	0.64	− 0.76

**Figure 2:**
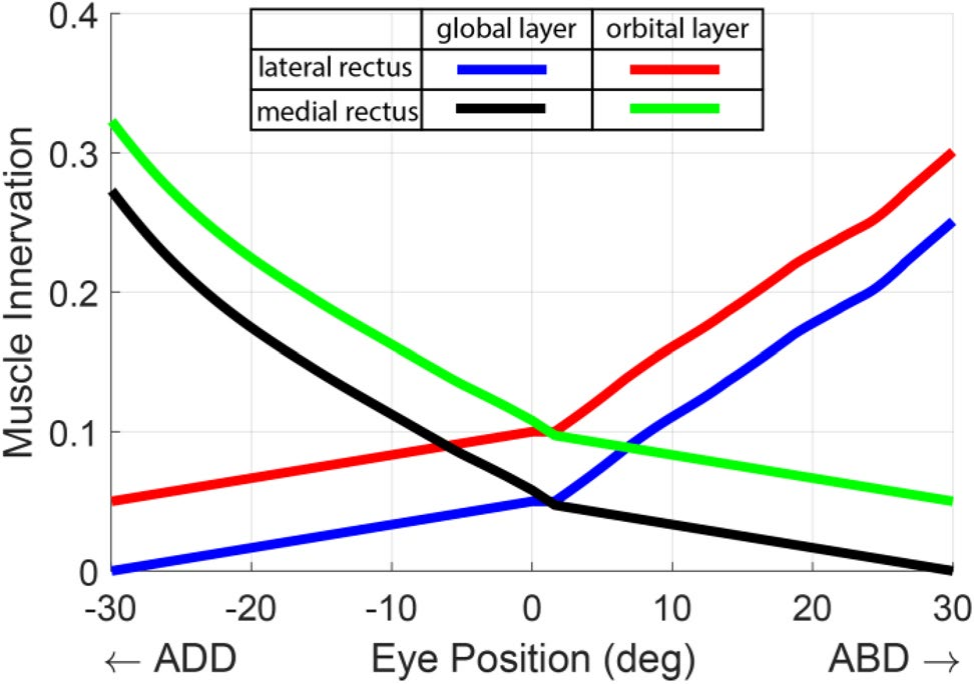
Innervations of lateral and medial rectus muscles at different horizontal eye positions during simulated fixation. Innervation is a unitless scalar between 0 and 1.

Figure 2 shows computed LR and MR innervations as functions of horizontal eye position between 30^o^ adduction and 30° abduction. By constraint consistent with human electromyographic data, the OL has greater innervation than GL throughout the field of action of each EOM. Innervations of OL and GL progressively increased as the eye rotates in the ON direction of the corresponding EOM. The relationships between innervation and eye position was roughly parabolic rather than linear.

Similar roughly parabolic curves for OL and GL innervations as functions of eye positions based on electromyography were reported by Collins^17^, who attributed the parabolic relationship to staggered fiber recruitment. This consistency between the EOM innervation profiles computed from the neural controller shown in Fig. 2 and the electromyographic measurement of GL and OL fiber innervations from human medial rectus muscle during fixation and slow eye movement^17^ demonstrates our model’s realism, which otherwise might have required non-physiologic innervation. Both OL and GL remain active during fixation far outside the field of action of the corresponding EOM, which was also observed experimentally^17^.

**Figure 3:**
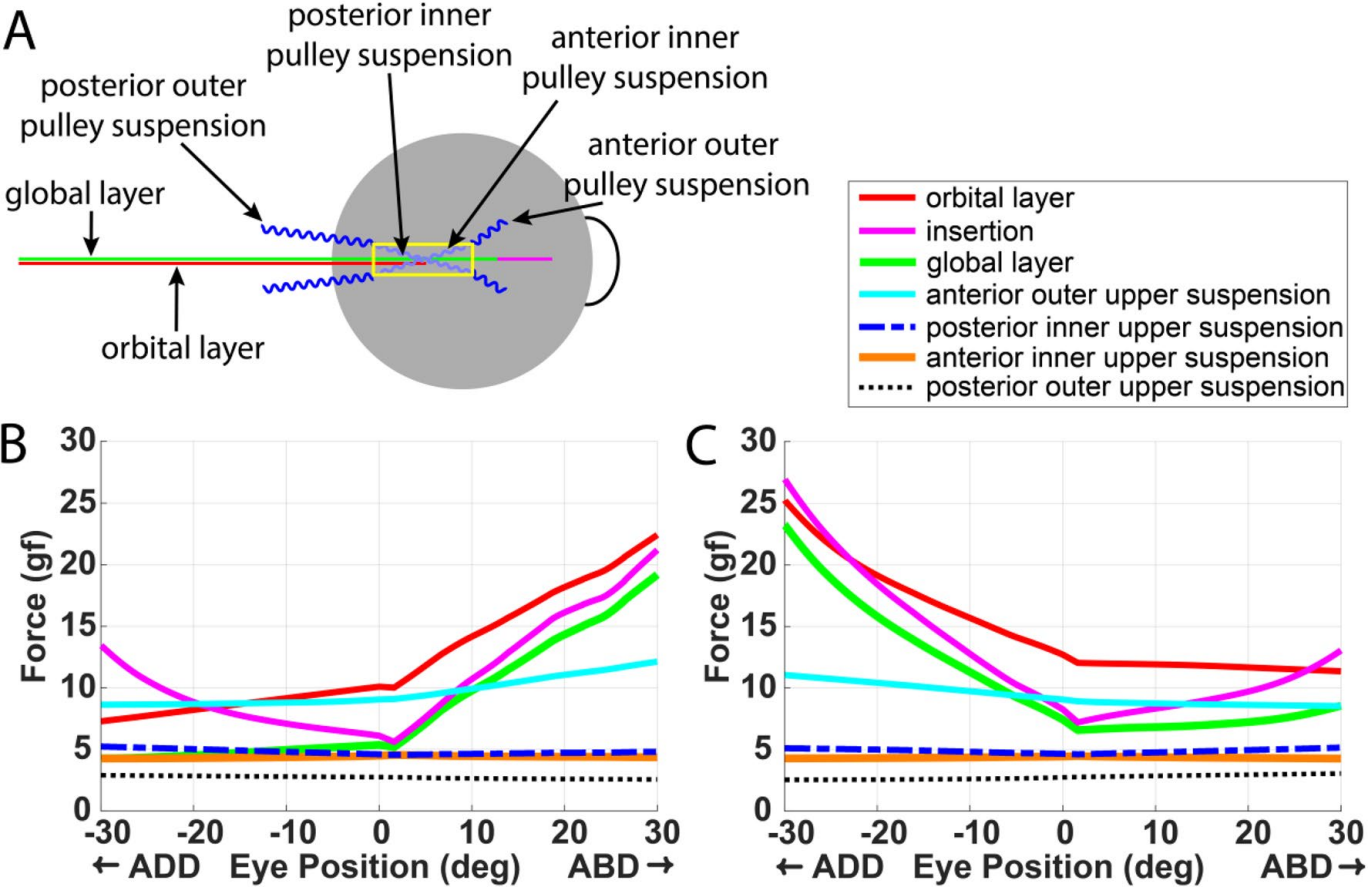
(**A**) Diagram showing EOM layers and suspensions. Simulated tensions of (**B**) lateral and (**C**) medial rectus muscles and their associated pulley suspensions at different horizontal eye positions.

Simulated LR and MR tensions as functions of horizontal eye position are shown in Fig. 3. During progressive rotation from 30° add- to 30° abduction, simulated MR insertional force corresponded with empirical measurements on unrestrained eye movement^26^, both in pattern and tension magnitude. MR force was about 25 g at 30° adduction, decreased as the eye moved temporally. After reaching its minimum, MR tension at its insertion increased to reach 10 g at 30° abduction. A similar relationship was observed for force at the LR insertion. The force increase at an EOM’s insertion in its extreme inhibited field of action is probably be caused by the antagonist EOM’s passive elastic force and elastic pulley suspension forces. Both MR OL and GL tensions increased in MR’s field of action as the OL applied significantly greater tension than GL, demonstrating different mechanical actions of OL and GL in fixation. Pulley suspension forces were read out from the simulator. Only the four superior suspensions are shown in Fig. 3. The four inferior suspension forces were identical to their corresponding upper suspensions during horizontal gazes. Pulley suspension forces ranged between 3 and 13 g, all varying with eye position. The anterior superior suspension applied the greatest force and thus contributed the most to stabilizing the pulley tubes transversely. Simulation of horizontal and vertical eye movement was provided in the supplementary video.

**Figure 4:**
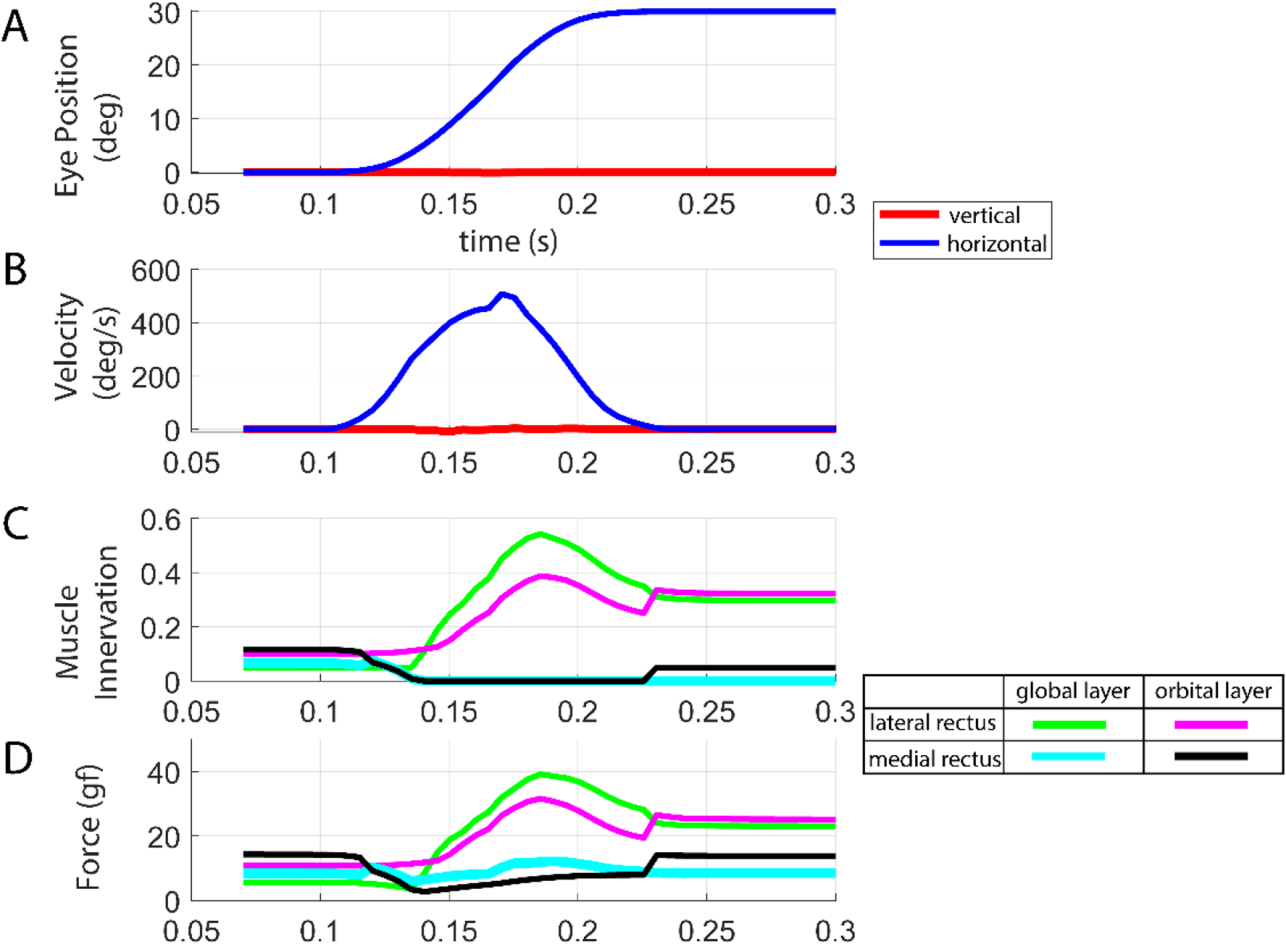
Simulation of a 30° saccade from central gaze to abduction: (**A**) eye position, (**B**) velocity, (**C**) innervation of lateral and medial rectus muscle, and (**D**) muscle tension.

Horizontal saccades were simulated. Figure 4 simulates a 30° saccade from central gaze to abduction. Simulated innervations and tensions of the LR, which is the agonist for this saccade, showed the expected pulse and step pattern. Innervation of the LR_OL_ was higher than that of the LR_GL_ during the main course of this saccade, which is consistent with the recordings of electromyographic activities in the GL and OL of an agonist human EOM^17^, and demonstrates differential laminar functions. The neural controller supplied zero innervation to the antagonist MR, again consistent with experimental findings that an antagonist EOM receives no innervation during saccades exceeding 10° ^17,27^. The simulated MR_GL_ tension showed a small peak despite zero innervation to the antagonist muscle during that period. This phenomenon demonstrated here has been observed empirically and has been explained by fast elongation during a high speed saccade^17^. The emergent ability of the new model to simulate the above specific mechanical characteristics of EOMs manifests the model’s realism and fidelity to empirical findings.

## Discussion

We have presented here a novel bilayer biomechanical model that realistically implements mechanical behavior of actively-controlled pulleys in secondary gazes. The model was verified against experimental data showing consistency in terms of EOM innervation, force and path when compared to measurement from human subjects reported in literature. Our model demonstrates differential control of OL and GL tensions and the physical plausibility of the actively controlled pulleys and their interactions with OL and GL of rectus EOMs in implementing fixation and saccadic eye movements. We conclude that Active Pulley Hypothesis is mechanically plausible on the horizontal rectus muscles.

Ocular motor modeling continues to play an important role in understanding mechanics and neural control of eye movement ^28^, but it has principally been failures rather than successes of modeling that have motivated progress in understanding the ocular motor plant. Unrealistic predictions of Robinson’s first 3D computer model of the orbit in 1975 inspired Joel Miller’s MRI study that motivated discovery of the pulley connective tissues^2^, revolutionizing our understanding of orbital anatomy and EOM structure and function. Several models of oculomotor biomechanics were developed subsequently but suffer from critical limitations and failures that motivated further anatomical and functional discoveries in the ocular motor system. Neurophysiology of nuclear and supranuclear ocular motor brainstem control centers cannot advance rationally without a mechanical model of the way that EOMs actuate ocular rotations. We plan to augment the current model to incorporate the pulley mechanics of the vertical EOMs to enable study of tertiary gazes.

The model proposed here is the first to explicitly simulate dynamic behaviors of the OL and GL of horizontal rectus extraocular EOMs. Although most previous biomechanical studies of the ocular plant have chiefly considered its behavior in fixation, simulating non-fixational eye movement can be informative to understand the compartmental architecture of the EOMs and how such a structural design might influence precision and stability. Such dynamic simulation can also be useful in clinical interpretation of rectus EOM palsy, where the dynamics of saccades and smooth pursuit might someday allow clinicians to distinguish the relatively common compartmental palsy from partial complete palsy.

## Methods

### Modeling global layer and orbital layer of the horizontal rectus muscles

Lateral rectus and medial rectus muscles are each represented by three musculotendon strands as defined in our previous papers^25^. A strand is a computational modeling primitive that represents the geometry and biomechanical characteristics of a part of the musculotendon aligned with the fibers. Each strand is a cubic B-spline curve associated with mass and constitutive models. Strands have been used to simulate muscle biomechanical functions^25,29^. The GL and OL are individually modeled by two contractile-elastic strands, while the EOM tendon is modeled by a non-contractile elastic strand. Each of the vertical and superior oblique EOMs is represented by a single layer contractile-elastic strand in series with a non-contractile elastic strand. The inferior oblique EOM is modeled by a single layer contractile-elastic strand. Insertion positions on the globe, and tendon lengths, were taken from published anatomical measurements^30^.

The three-element Hill type muscle constitutive model was used for the EOM strands. The total force of a muscle strand ${F}^{muslce}$ was a function of its activation $a\in \left[\mathrm{0,1}\right]$, length ${l}^{muscle}$ and velocity ${v}^{muscle}$. Muscle force ${F}^{muslce}$ was computed as the sum of the active force and the passive force: ${F}^{muslce}={F}^{active}+{F}^{passive}=a\cdot FL({l}^{muscle} )\cdot FV({v}^{muscle})+{F}^{passive}$. $FL({l}^{muscle} )$ and $FV({v}^{muscle} )$ are nonlinear force–length and force–velocity functions and more details about EOM strand mechanics can be found in our previous paper^25^. The globe was assumed spherical and constrained to rotate about its geometric center.

### Pulley modeling

During head-fixed eye rotation without vergence, even strabismic eye movements robustly conform to Listing’s law, which specifies uniqueness of torsion for any combination of horizontal and vertical eye orientation^30–32^. In order to enable the ocular plant to implement Listing’s law in fixation^13^, the APH implies that a realistic active pulley model must possess the following characteristics. The net force on the pulley transverse to the EOM path direction should be sufficiently large to permit very little EOM sideslip for all secondary and tertiary eye positions. The net longitudinal force on the pulley should be sufficiently small to be compatible with physiologic EOM tension over the full range of contraction and relaxation. The APH proposes that anteroposterior pulley position is regulated by the balance of tensions in the OL and pulley suspensions, so that the pulley can serve as the functional origin of each rectus EOM.

We initially experimented with several pulley architectures by varying the number of pulley suspension strands, their orientations and lengths, as well as their mechanical couplings among the associated rectus EOMs. The essential characteristic of pulleys is that they limit (not eliminate) muscle sideslips. Two opponent pulley suspensions are the most concise architecture to mechanically accomplish such function therefore we started with two pulley suspension strands. As more components being added during model improvement, it became clear that more pulley suspensions were needed. The final model included 8 pulley suspensions, which is the minimal set to implement the coupling among EOM layers, orbital wall and pulley tube as well as to result in realistic horizontal rectus EOM contraction/elongation in both transverse and longitudinal direction. We kept the model as simple as possible to avoid an “overfitting” model with unnecessary complexity that would have defeated our purpose to understand the essential underlying mechanism of extraocular muscle mechanics.

**Figure 5:**
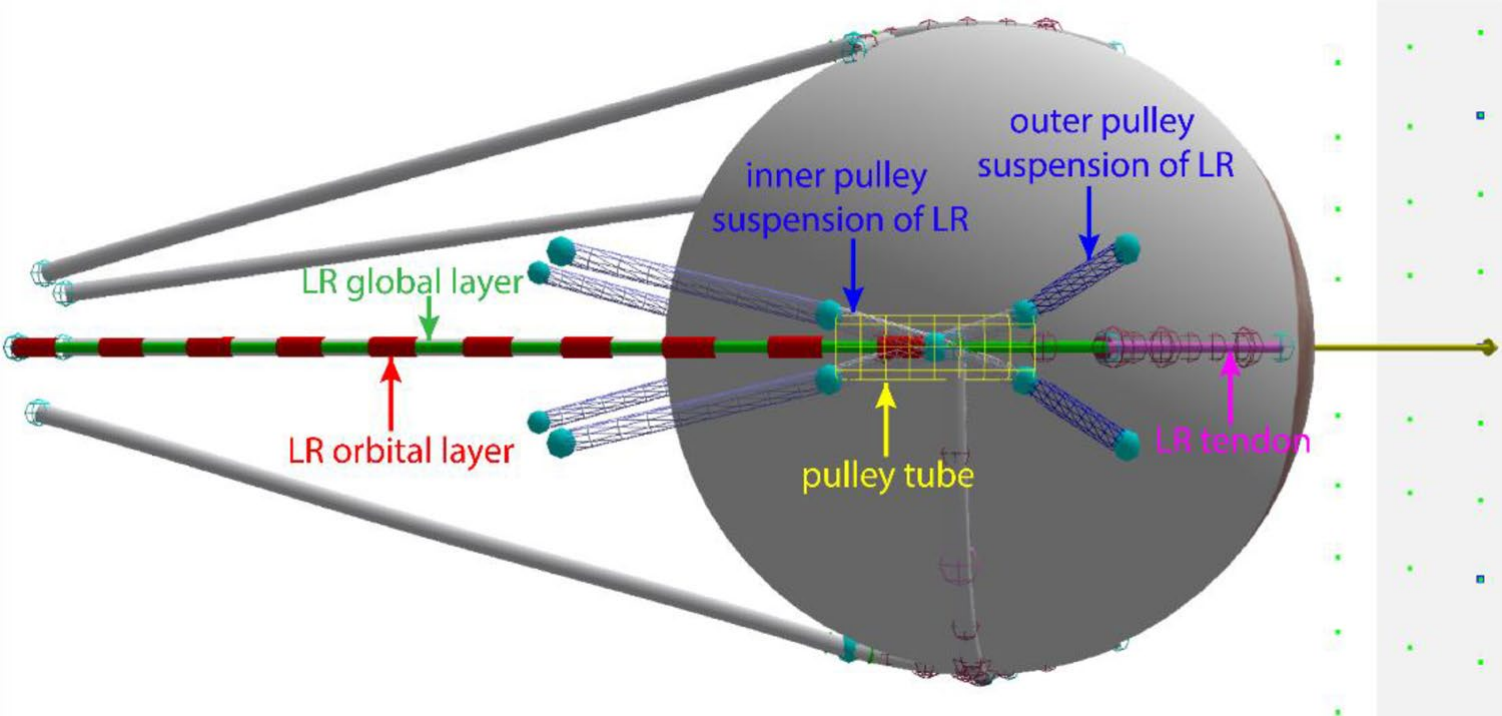
A lateral view of the new pulley model. The global layer and orbital layer of the lateral extraocular muscle are modeled as two separate muscle strands, as also implemented for the two layers of the medial rectus muscle that is not seen in this view. A pulley tube system was developed to model active pulley mechanics. LR: lateral rectus muscle.

In this new model that we term the “pulley tube system,” 8 strands supporting a tubular ensheathment of each horizontal rectus EOM were used to model the pulley. Figure 1 shows the pulley tube system associated with the LR muscle. The pulley is modeled by a rigid tube to which the OL is inserted. This pulley tube works as a common junction for the OL, GL, and all pulley suspensions. Position of the pulley tube was evaluated as a criterion for model performance to correspond with pulley positions empirically measured by MRI in human adults^13^. The pulley tube is associated with a geometric mesh. Surface contact constraint is enforced between the GL and pulley tube surface to confine the GL within the tube. This constraint ensures that the GL slides through the pulley tube with no more than permitted transverse movement, and never strays outside the tube, which is a physiological impossibility. The pulley tube is supported by four suspensions, referred as the outer pulley suspensions in Fig. 5. Each pulley suspension is modeled as a non-contractile elastic strand, similar to a tendon strand. These outer pulley suspensions have fixed origins (on the orbital wall) and insert on the pulley tube, contributing to proper positioning of the pulley. Four inner pulley suspensions were created to simulate the mechanical interaction between the GL and the pulley tube. These inner suspensions allow longitudinal movement of the GL relative to the pulley tube while restricting transverse movement. Stiffnesses, orientations, and origin locations of the outer suspensions were determined empirically to limit side-slip of OL and GL while allowing anteroposterior travel of OL and GL.

### Neural controller

We employed the neural controller in our previously developed orbit model to implement EOM innervations^25^. The neural controller^25^ was formulated to find the set of innervations of the minimal summed muscle innervation for a given dynamic eye movement. No assumption was made explicitly on the reciprocal innervations of the six EOMs, which were treated as independent actuators of the ocular plant. In this study, we computed the innervation set $A$ needed for the new model both to fixate the eye at a particular eye position, and also to simulate saccadic eye movement. The 8 variables in $A$ include unitless innervations of the LR global layer (LR_GL_), LR orbital layer (LR_OL_), MR global layer (MR_GL_), MR orbital layer (MR_OL_), superior rectus muscle (SR), inferior rectus muscle (IR), superior oblique (SO) muscle, and inferior oblique (IO) muscle: $A=[{a}^{{LR}_{GL}},{a}^{{LR}_{OL}},{a}^{{MR}_{GL}},{a}^{{MR}_{OL}}{,a}^{SR},{a}^{IR},{a}^{SO},{a}^{IO}]$. Innervation set $A$ is solved by minimizing the following cost function consisting of the weighted sum of the total EOM innervation ${\Vert A\Vert }^{2}$ and eye velocity error which is the difference between eye velocity ${v}^{eye}$ and target velocity ${v}^{target}$:

1 $$\underset{A}{\mathrm{min}}{ w}_{a}{\cdot \Vert A\Vert }^{2}+{w}_{x}{\cdot \Vert {v}^{eye}-{v}^{target}\Vert }^{2}.$$

${w}_{a}$ and ${w}_{x}$ are the blending weights of the two terms so that both the summed innervation and velocity error are minimized. The neural controller was formulated in velocity space and more details can be found in our previous paper^25^. To simulate static fixation, zero target velocity was applied at the target position. Two additional constraints were added to enforce greater OL than GL innervation during MR and LR contraction. Differential innervation was set to be 0.05 to approximate the greater OL than GL electromyographic activities measured by Collins in humans^17^. Such constraints were not enforced between onset and offset of simulated saccades, which were defined as globe velocity of at least 20°/s.

## Supplementary Information

Supplementary Video 1.

## Data Availability

Recordings of simulated eye movement were submitted as supplementary materials. Simulation results are available from the corresponding author on reasonable request.
